# Identification of multiple isoforms of glucocorticoid receptor in nasal polyps of patients with chronic rhinosinusitis

**DOI:** 10.1186/s40463-022-00561-1

**Published:** 2022-06-11

**Authors:** Shan Shao, Yue Wang, Yan Zhao, Yuan Xu, Tie Wang, Kun Du, Shiping Bao, Xiangdong Wang, Luo Zhang

**Affiliations:** 1 Department of Otorhinolaryngology Head and Neck Surgery, Beijing Tongren Hospital, Capital Medical University, Beijing, 10073 China; 2 Department of Otolaryngology Head and Neck Surgery, Beijing Youan Hospital, Capital Medical University, Beijing, 100069 China; 3 Beijing Key Laboratory of Nasal Diseases, Beijing Institute of Otolaryngology, No. 17, Hougou Hutong, Dong Cheng District, Beijing, 100005 China; 4 Departments of Oncology, Community Health Sciences, and Surgery, Cumming School of Medicine, and The Center for Health Informatics, University of Calgary, Calgary, AB Canada; 5 MIID Snyder Institute for Chronic Diseases, Cumming School of Medicine, University of Calgary, Calgary, AB Canada

**Keywords:** Nasal polyps, Sinusitis, Glucocorticoid receptor, Endotypes

## Abstract

**Background:**

The conventional belief that glucocorticosteroid (GC) acts through a single brand glucocorticoid receptor (GR)α protein has changed dramatically with the discovery of multiple GR isoforms. We aimed to evaluate whether multiple GR protein isoforms are expressed in chronic rhinosinusitis with nasal polyps (CRSwNP) and whether GR protein isoform expression profiles differ between different endotypes of CRSwNP.

**Methods:**

Thirty-eight patients with CRSwNP and ten healthy volunteers were included. The protein expression of multiple GR isoforms in nasal polyps (NPs) tissue and control mucosae was examined by western blot analysis with different GR antibodies.

**Results:**

Five bands, including three bands for known proteins (GRα-A/B, GRα-C, and GRα-D) and two bands for unidentified proteins at 67 kilodaltons (kDa) and 60 kDa, were identified with both total GR antibody (PA1-511A) and GRα-specific antibody (PA1-516). GRα-D intensity, which was abundant in nasal mucosa, was significantly increased in the CRSwNP group and was especially elevated in the noneosinophilic CRSwNP (NE-CRSwNP) group (PA1-511A: *P* < 0.001 and *P* = 0.0018; PA1-516: *P* < 0.003 and *P* = 0.006, respectively). Additionally, the intensities of the newly recognized 67 kDa and 60 kDa bands were much greater in the NE-CRSwNP subgroup than in the eosinophilic CRSwNP (E-CRSwNP) subgroup; in the E-CRSwNP subgroup, the median intensities were even lower than those in the control group.

**Conclusions:**

This study provides evidence that nasal tissues express multiple GR protein isoforms. GR protein isoforms presented disease and tissue-specific expression profiles that differed between the CRSwNP and control groups and between the E-CRSwNP and NE-CRSwNP subgroups.

**Graphical abstract:**

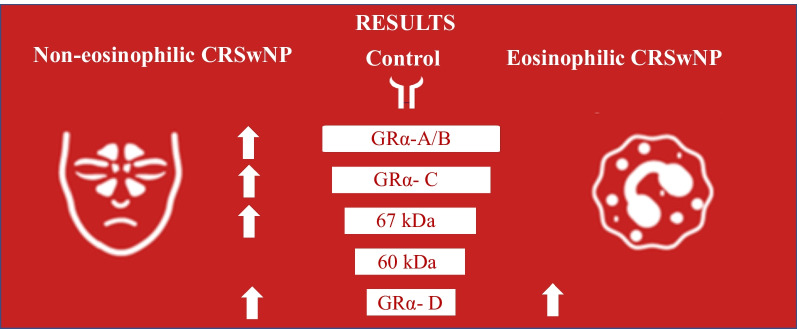

**Supplementary Information:**

The online version contains supplementary material available at 10.1186/s40463-022-00561-1.

## Background

Chronic rhinosinusitis (CRS), is a heterogeneous disease characterized by a defective immune barrier and massive inflammatory cell infiltration, affecting approximately 8% in China, 5% in Canada, and up to 10–12% in the United Kingdom and the United States, which causes increasing healthcare costs, annual physician visits, and significant decreases in workplace productivity [1–4]. A growing body of evidence suggests important heterogeneity in both clinical phenotypes and inflammatory endotypes of patients with CRS [5]. CRS with nasal polyps (CRSwNP) can be further classified into two endotypes based on the degree of eosinophil infiltration into the nasal mucosa: eosinophilic CRSwNP (E-CRSwNP) and noneosinophilic CRSwNP (NE-CRSwNP) [6, 7]. Compared with NE-CRSwNP, E-CRSwNP has been shown to be frequently associated with a higher incidence of asthma, increased sensitivity to glucocorticoid therapy, and higher recurrence rate [6]. Glucocorticosteroid (GC), is recommended as the first-line medicine for the treatment of chronic rhinosinusitis with nasal polyps (CRSwNP) [7]. As the biological effects of GC are mediated primarily through activation of intracellular glucocorticoid receptors (GR), recent evidence suggests that altered GR isoform expression may be associated with different GC sensitivity and disease severity [8–11].

Recently, the conventional belief that GC acts through a single 94 kilodaltons (kDa) GRα protein has changed dramatically with the discovery of multiple GR isoforms [12]. GR could generate at least 5 characterized splice variants (GRα, GRβ, GRγ, GRA, and GRP) through alternative splicing. Via alternative initiation of translation from one single species of mRNA, GRα could further generate eight translational isoforms (A, B, C1, C2, C3, D1, D2, and D3) [11, 13–17]. Moreover, different GR isoforms generated by alternative splicing and translation initiation have different functions [12, 13, 18–20]. GRα-C isoform has been demonstrated to confer increased susceptibility to apoptosis in cell culture, while GRα-D isoform confers relative resistance to apoptosis under the same conditions [12, 21]. GRβ, which is expressed at much lower levels than GRα, was reported to be associated with GC resistance in some inflammatory disorders, but the results have been controversial and need further research [21–23]. In addition, the three newly recognized splice variants GRγ, GR-A and GR-P have also been implicated in GC resistance cancer cells [16, 18]. Overall, the evidence indicates that GC actions may not be mediated only by the well-characterized GRα-A; rather, it may involve a far more complex process with several GR isoforms that are coexpressed and potentially interact with one another [24].

In the past thirty years, many studies have been devoted to studying GC sensitivity and disease severity in CRS from the perspective of GR. However, previous studies on GR protein isoforms have focused mainly on the single strand 94 kD GRα-A and/or 91 kD GRβ by western blot (WB) analysis or immunohistochemical or immunofluorescence methods, and the results have been controversial and inconsistent [22, 25, 26]. We speculate that the inconsistency of the results could be, to some extent, that GR, especially the predominant GRα, could further generate multiple protein isoforms, which exhibited different functions in tissue- and disease-specific manner.

Therefore, it is necessary to re-evaluate the expression profiles of multiple GR protein isoforms in the context of tissue- and disease-specific conditions. However, the expression profiles of multiple GR translational isoforms have yet to be studied in nasal tissues. The current study aimed to 1) examine whether human NPs and control nasal mucosae express multiple GR isoforms and 2) determine whether GR isoform expression profiles differ between patients with CRSwNP and control subjects as well as between different endotypes of CRSwNP.

## Patients and methods

### Patients

A total of 48 subjects, including 38 patients with CRSwNP and 10 control subjects undergoing septoplasty because of anatomic obstruction, were involved retrospectively from the Rhinology Department of Beijing TongRen Hospital between January 2017 and December 2019. Nineteen eosinophilic and nineteen noneosinophilic NP tissue samples were collected from patients with CRSwNP undergoing functional endoscopic sinus surgery. Uncinate process mucosa were obtained as control tissue samples from the control subjects during surgery of septoplasty. NP and uncinate mucosae tissue samples were divided into specimens; one was snap-frozen in liquid nitrogen and stored at − 80 °C for WB analysis, the other was fixed in paraformaldehyde for hematoxylin and eosin staining. The authors are accountable for all aspects of the work in ensuring that questions related to the accuracy or integrity of any part of the work are appropriately investigated and resolved. The study was conducted in accordance with the Declaration of Helsinki (as revised in 2013). The study was approved by the Ethics Committee of Beijing TongRen Hospital (TREC 2011–21), and individual consent for this study was obtained.

The diagnosis of CRSwNP was made based on each patient’s history, clinical manifestation, and results of nasal endoscopy and computed tomographic scanning according to the EPOS 2012 guidelines [27]. The exclusion criteria were as follows: (1) age younger than 18 years; (2) history of receiving treatment with antibiotics, systemic or topical corticosteroids, or other immune-modulating drugs within the previous 4 weeks prior to surgery; and (3) diagnosis with unilateral rhinosinusitis, antrochoanal polyp, allergic fungal rhinosinusitis, cystic fibrosis, or immotile ciliary disease. None of the subjects used specific medications (oral or local corticosteroids, antihistamines, leukotriene receptor antagonist and antibiotics) within 4 weeks before sample collection [6]. All subjects were free of upper respiratory tract infections for the 4 weeks preceding the study. The diagnosis of allergic rhinitis (AR) was based on the current recommendations [28]. Atopic status was evaluated by measuring serum-specific IgE (Phadia, Uppsala, Sweden) against various common inhalant allergens (house dust mites, molds, trees, weeds, grass pollen, and animal dander). The diagnosis of asthma was made according to identifying both a characteristic pattern of respiratory symptoms such as wheezing, shortness of breath, chest tightness or cough, and variable expiratory airflow limitation [29].

### Hematoxylin and eosin staining

Samples were fixed in 4% paraformaldehyde and then dehydrated in 95% ethanol. After clearing and embedding, the samples were sectioned at 4 μm thickness and stained with hematoxylin and eosin (H&E) according to the manufacturer’s instructions to assess infiltration by different types of inflammatory cells. Two independent observers who were blinded to the diagnoses and clinical data counted the positive cells, including eosinophils, neutrophils, plasma cells and lymphocytes, in 10 randomly selected high-power fields (HPFs) by brightfield light microscopy (BX51, Olympus, Japan) at × 400 magnification. Eosinophilic CRSwNP (E-CRSwNP) was defined by the presence of more than 10 eosinophils per HPF in an NP, while noneosinophilic CRSwNP (NE-CRSwNP) was defined by the presence of fewer than 10 eosinophils per HPF in an NP [7, 30, 31].

### WB analysis

Nasal tissues were cut into small pieces with a sterile surgical blade and homogenized in liquid nitrogen. The homogenates were lysed in radioimmunoprecipitation assay buffer (Solarbio, Beijing, China) containing 1 mM phenylmethylsulfonyl fluoride (Solarbio, Beijing, China). The protein concentrations were determined with a bicinchoninic acid assay kit (Beyotime, Beijing, China). The proteins were immediately heated for 5 min at 95 °C and then the protein fractions (10–20 µg) subjected to sodium dodecyl sulfate–polyacrylamide gel electrophoresis on gels containing 8% (w/v) acrylamide under reducing conditions. The separated proteins were transferred to nitrocellulose membranes (Abclonal, Wuhan, China), and then the membranes were blocked with 5% skim milk [32]. Commercial WB analysis was performed using a ChemiDoc™ Imaging System (Bio-Rad, USA). The following antibodies were used for WB analysis: anti-GRα (1:1000, Invitrogen, PA1-516), anti-GR (1:1000, Invitrogen, PA1-511A), anti-β-actin (1:10,000, Sigma, A5441), goat anti-mouse (1:10,000, Multi Sciences, GAM007), and goat anti-rabbit (1:10,000, Multi Sciences, GAR007). The band densities of the target proteins were estimated with Image Lab image analysis software (Bio-Rad Laboratories). All receptor isoform values were normalized to β-actin expression. To compare the levels among bands, protein expression was further analyzed relative to the expression of an internal control [16]. In addition, the protein concentration for each band was normalized to the total GR expression (sum of GR bands 1–5 for a single group), which allowed comparisons to be made between GR isoforms within a sample [33].

### Statistics

GraphPad Prism software 8.0 (GraphPad Software Inc., San Diego, CA, USA) was used for all statistical analyses. Continuous variables are expressed as the mean ± the standard error of the mean (SEM). GR isoform data are presented as the median and interquartile range (IQR) values or as ratios to the total GR content identified by the same GR antibody. The GR data were not normally distributed, so nonparametric tests (Mann–Whitney tests) were used. Pearson's correlation analyses were performed to assess associations between two groups. Categorical variables are presented as frequencies (%) and were compared using the χ^2^ test or Fisher’s exact test between groups. *P* < 0.05 was considered to indicate statistical significance.

## Results

### Clinical characteristics of patients with CRSwNP and control subjects

The clinical characteristics of patients with different endotypes of CRSwNP and control subjects are shown in Table 1. There were no significant differences among the groups in terms of age, sex, smoking history, prior sinus surgery, allergic rhinitis.

**Table 1:** Clinical characteristics of individuals in the CRSwNP and control groups

Characteristic	Control (N = 10)	Eosinophilic CRSwNP (N = 19)	Noneosinophilic CRSwNP (N = 19)	*P*-value*
Age (years), mean ± SD	47.1 ± 15.5	49.26 ± 11.0	49.16 ± 14.1	0.905
Sex, male, n (%)	6 (60)	11 (57.9)	16 (84.2)	0.155
Smoking, n (%)	3 (30)	2 (10.5)	5 (26.3)	0.329
Prior sinus surgery, n (%)	0 (0)	6 (31.2)	4 (21.1)	0.053
Allergic rhinitis, n (%)	0 (0)	4 (21.1)	3 (15.8)	0.153
Asthma, n (%)	0 (0)	5 (26.3)	1 (5.3)	0.040*

*CRSwNP* chronic rhinosinusitis with nasal polyps, *SD* standard deviation

*P < 0.05. The P-values indicate the differences between patients with CRSwNP and control subjects, and P-values less than 0.05 were considered to indicate statistical significance

### Detection of multiple GR isoforms in the CRSwNP group and control group

**Fig. 1:**
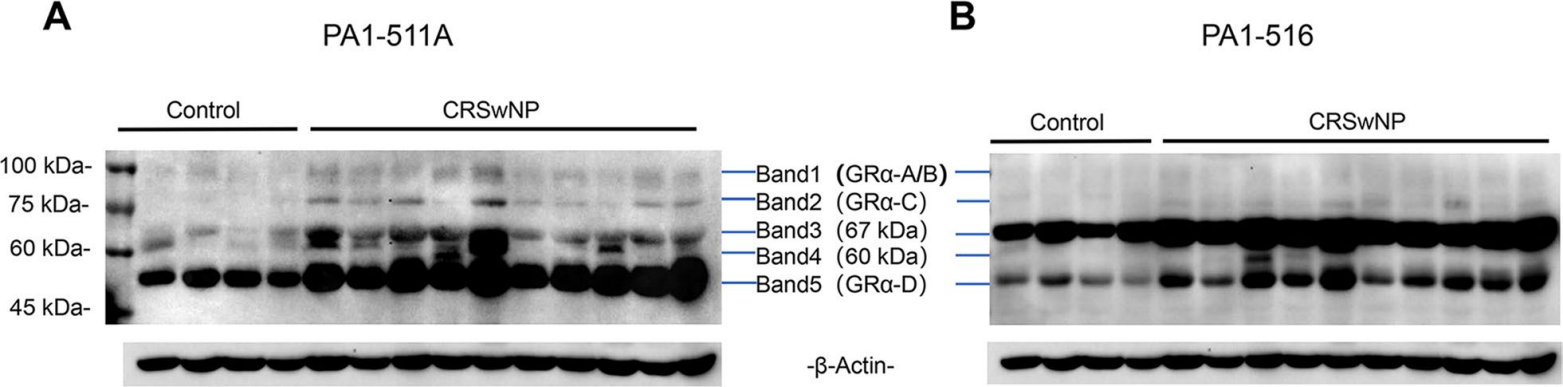
Western blots of representative GR bands in the CRSwNP group and control group. NP tissues and control mucosae were probed with **a** an anti-total GR antibody (PA1-511A) and **b** an anti-GRα antibody (PA1-516). The images showed five GR bands of approximately 91–94, 80, 67, 60 and 52 kDa. β-Actin was used as an internal control. *GR* glucocorticoid receptor, *CRSwNP* chronic rhinosinusitis with nasal polyps, *kDa* kilodaltons

In total, five bands with approximate MWs of 91–94, 80, 67, 60 and 52 kDa in NP tissue and control mucosae were identified by WB analysis using a specific anti-GRα antibody and an anti-total GR antibody, whose episodes correspond to distinct peptide segments on the hGRα, respectively (Fig. 1). Since the 94 kDa and 91 kDa bands were not easy to distinguish in some individuals, we combined the two bands as an integrated band. According to previous studies of multiple GR isoforms identification [13, 14, 33], we established that band 1 corresponded to GRα-A/B, band 2 corresponded to GRα-C, band 3 represented an uncharacterized 67 kDa isoform, band 4 represented an uncharacterized 60 kDa isoform, and band 5 corresponded to GRα-D. The relative expression intensity for each band was consistent between the two batches of GR antibodies, with positive correlations for the sum of bands 1–5, GRα-A/B and GRα-D (r = 0.7761, r = 0.6506, and r = 0.8690, all *P* < 0.0001) (Supplementary Fig. S1). We also found that both GR antibodies exhibited very high affinity for GRα-D. The PA1-516 antibody displayed substantially greater affinity for 67 kDa than the PA1-511A antibody, whereas the PA1-511A antibody presented greater affinity for the GRα-A, GRα-C and 60 kDa isoforms than the PA1-511A antibody. Because GRα-C, 67 kDa and 60 kDa isoforms exhibited different affinities with the two GR antibodies, the intensities of the corresponding bands were calculated with the associated higher affinity GR antibody.

### Expression profiles of multiple GR isoforms in the CRSwNP and control groups

**Fig. 2:**
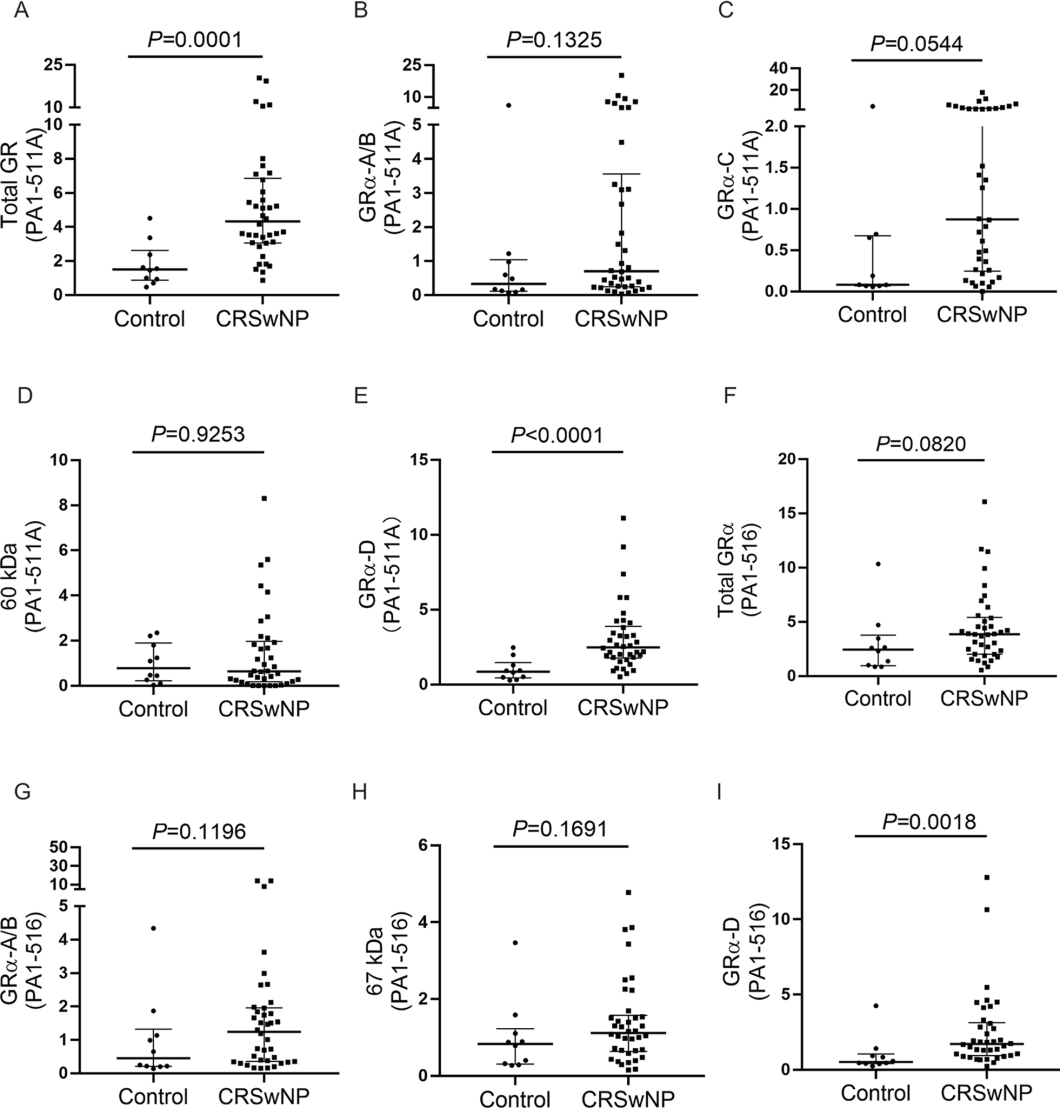
Expression profiles of representative GR isoforms between the CRSwNP group and the control group. The relative expression levels of **a** the sum of bands 1–5 and representative bands for GRs, including **b** GRα-A/B, **c** GRα-C, **d** the 60 kDa protein and **e** GRα-D, which were detected with an anti-total GR antibody (PA1-511A), were analyzed in the CRSwNP group compared to the control group. Similar band intensity comparisons were performed to analyze **f** the sum of bands 1–5 and the bands for **g** GRα-A/B, (H) the 67 kDa protein, and **i** GRα-D, which were detected with an anti-GRα antibody (PA1-516). Data was presented as a ratio of each isoform to an internal control which were relative to β-actin expression. The data were presented in dot plots with medians and interquartile ranges. Nonparametric tests (Mann–Whitney tests) were used for comparisons between these two groups. GR, glucocorticoid receptor; CRSwNP, chronic rhinosinusitis with nasal polyps; kDa, kilodaltons

The relative expression levels of multiple GR isoforms were compared between the CRSwNP and control groups with two GR-specific antibodies (Fig. 2). No significant differences in the intensities of the GRα-A/B, GRα-C and 60 kDa bands were observed between the CRSwNP group and the control group. However, the GRα-D band intensity was significantly higher in the CRSwNP group than in the control group (PA1-511A: 2.5; 1.8–3.9; *P* < 0.0001; PA1-516: 1.7; 0.9–3.1; *P* = 0.0018). The sum of the relative intensities of bands 1–5 detected with PA1-511A was significantly higher in the CRSwNP group than in the control group (P = 0.0001), whereas no significant difference was observed in the sum of the intensities of bands 1–5 detected with the PA1-516 antibody (P = 0.0820).

### Multiple GR protein profiles in different endotypes of CRSwNP and control groups

**Fig. 3:**
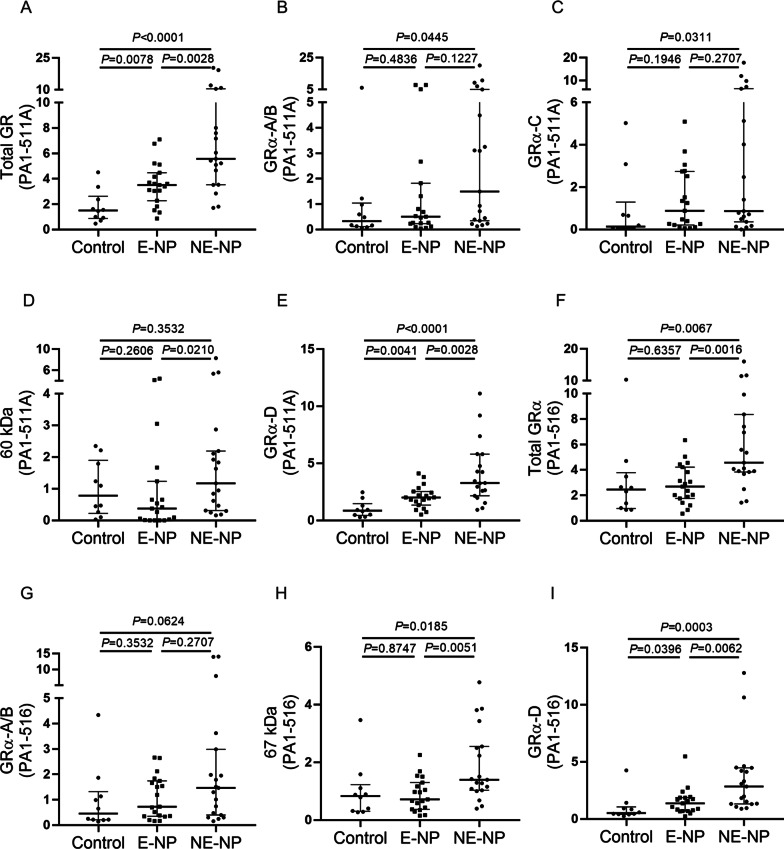
Expression profiles of representative GR isoforms among subgroups representing different endotypes of CRSwNP and the control group. The relative expression levels of **a** the sum of bands 1–5 and representative bands for GRs, including **b** GRα-A/B, **c** GRα-C, **d** the 60 kDa protein and **e** GRα-D, which were detected with an anti-total GR antibody (PA1-511A), were analyzed among the E-CRSwNP subgroup, the NE-CRSwNP subgroup and the control group. Similar band intensity comparisons were performed to analyze **f** the sum of bands 1–5 and the bands for **g** GRα-A/B, (H) the 67 kDa protein, and **i** GRα-D, which were detected with an anti-GRα antibody (PA1-516). Data was presented as a ratio of each isoform to an internal control which were relative to β-actin expression. The data were presented in dot plots with medians and interquartile ranges. GR, glucocorticoid receptor; CRSwNP, chronic rhinosinusitis with nasal polyps; kDa, kilodaltons; E-NP, eosinophilic nasal polyp; NE-NP, noneosinophilic nasal polyp

The relative levels of multiple GR isoforms were compared between different endotypes of CRSwNP and the control group with both GR antibodies (Fig. 3). No significant differences in the intensities of the GRα-A/B and GRα-C bands were observed between the NE-CRSwNP and E-CRSwNP subgroups. However, the GRα-D band intensities were dramatically increased in the NE-CRSwNP subgroup and the E-CRSwNP subgroup (PA1-511A: 3.3; 2.2–5.8; *P* < 0.0028; PA1-516: 2.8;1.3–4.5; *P* = 0.0062). In addition, the intensities of the unknown 67 kDa and 60 kDa bands were lower in the E-CRSwNP subgroup than in the NE-CRSwNP subgroup, and the median intensities were even slightly lower than those of the control groups. Similar results were obtained for the sums of the intensities of bands 1–5 detected with PA1-516 (*P* = 0.0016).

### Percent expression of GR isoforms in the CRSwNP group and control group

**Fig. 4:**
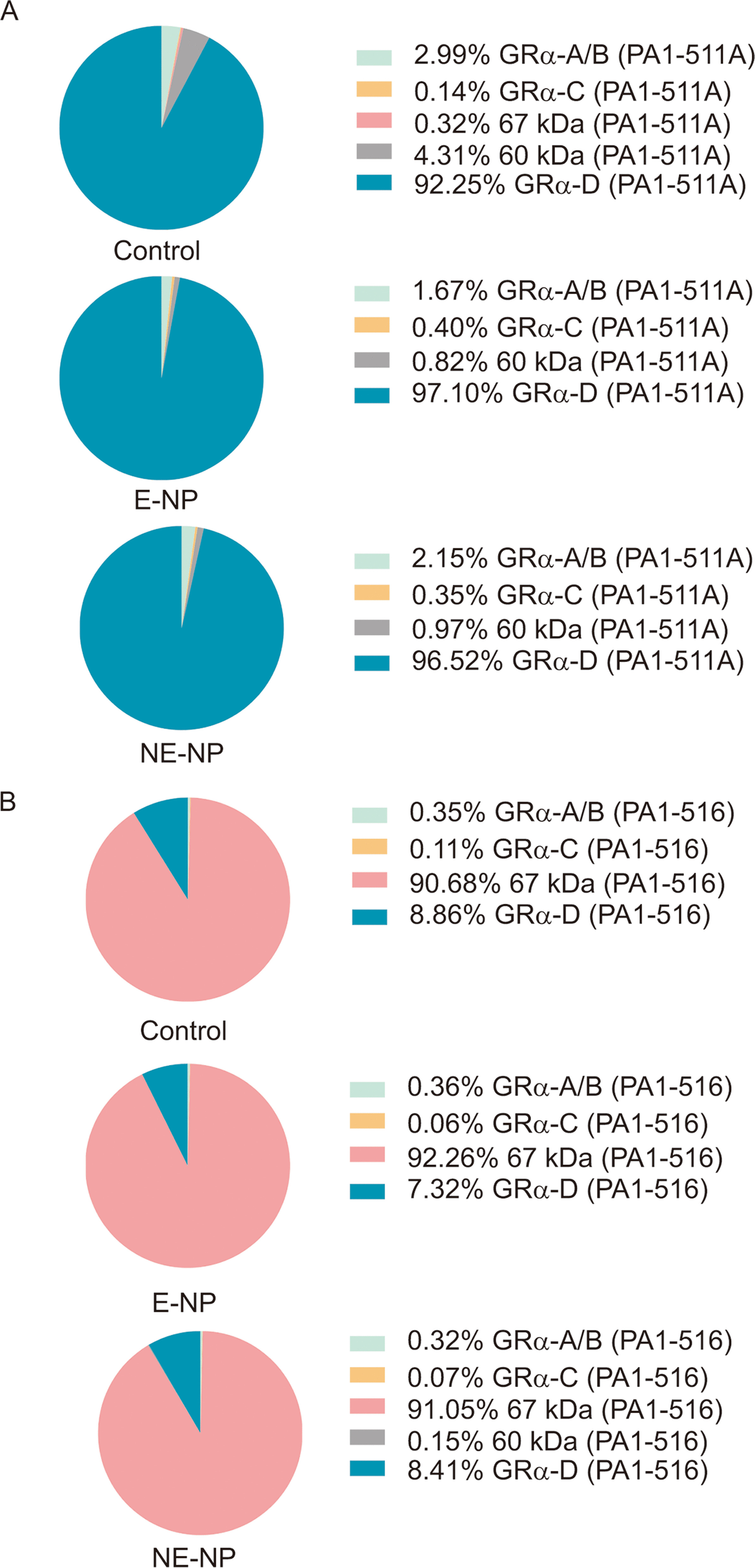
Percent expression of GR isoforms in subgroups representing different endotypes of CRSwNP and the control group. The isoforms were detected with **a** an anti-total GR antibody (PA1-511A) and **b** an anti-GRα antibody (PA1-516) in the subgroups representing different endotypes of CRSwNP and the control group. The percentages were calculated as the relative expression levels of individual GR isoforms compared to the sum of expression levels for bands 1–5. The pie charts displayed the medians for all individuals in either the different endotype subgroups of the CRSwNP group or the control group. GR, glucocorticoid receptors; CRSwNP, chronic rhinosinusitis with nasal polyps; kDa, kilodaltons; E-NP, eosinophilic nasal polyp; NE-NP, noneosinophilic nasal polyp

The percent expression of each band was calculated as the ratio over the sum for bands 1–5, and pie charts with the results are shown in Fig. 4. The anti-GRα antibody (PA1-516) revealed that the previously unknown 67 kDa protein was predominant in nasal tissues, accounting for 92.26% of the protein expression in the E-CRSwNP subgroup and 91.05% of that in the NE-CRSwNP subgroup (*P* = 0.6650). GRα-D was the second most abundant protein, accounting for 7.32% of the expression in the E-CRSwNP subgroup and 8.41% of the expression in the NE-CRSwNP subgroup (*P* = 0.6032). When PA1-511A was used for detection, the most abundant isoform was GRα-D (E-CRSwNP 97.10% vs. NE-CRSwNP 96.52%, *P* = 0.1542). Interestingly, compared to the other GR isoforms, classical GRα-A was weakly expressed, constituting less than 3% of the total expression (sum of the isoforms for bands 1–5) detected with both GR antibodies.

## Discussion

To our knowledge, this study is the first to demonstrate the presence of multiple GR isoforms in human nasal tissues with a combination of different GR antibodies. Our study found that at least five GR isoforms with MWs of approximately 91–94, 80, 67, 60 and 52 kDa exist in NPs and control mucosae in addition to the archetypal full-length 94 kDa GRα-A. According to research on multiple GR isoforms, we established that band 1 corresponded to GRα-A/B, band 2 represented GRα-C, and band 5 represented GRα-D. In addition, we also identified two previously unknown isoforms, the 67 kDa isoform and 60 kDa isoform, which were detected with both batches of GR antibodies. Importantly, we found that GR isoforms presented disease and tissue-specific expression profiles either between the CRSwNP and control groups or between the E-CRSwNP and NE-CRSwNP subgroups.

Recently, several translational isoforms of GR have been reported to exist in different species, various tissues and several cell lines [11, 13, 34, 35]. According to the previous study of Nick, when the human GRα was expressed in COS-1 cells, which lack endogenous GR, multiple proteins of 94, 91, 82–84, and 53–56 kDa were detected by using different selective anti-peptide GR antibodies [13]. In a series of Australian studies on the expression of multiple GR isoforms in different species with a total GR antibody, multiple known GR isoforms (such as GRα-A, GRα-C, GRα-D) and some unknown GR isoforms were identified in the placenta of humans, guinea pigs, mice and a sheep model of maternal allergic asthma [16, 24, 33, 34]. However, some variations in the expression patterns of GR isoforms have still been observed among species and tissues. For example, GRα-B is not expressed from the GR gene in guinea pigs because a second start codon on exon 2 of the guinea pig GR gene encodes isoleucine instead of methionine [24]. In another study on GR isoforms of human dorsolateral prefrontal cortex (DLPFC) detected with the GRα-specific antibody, five bands were identified by WB analysis and further verified in Hela cells by transfecting with GRα-A, GRα-C 1and GRα-D1variants [14]. Sinclair et al. concluded that band1 corresponded to GRα-A, the 67 kDa band represented a newly uncharacterized GR isoform, the 50 kDa band represented the truncated GRα-D1 isoform in human DLPFC tissue [14]. In our study, the previously identified isoforms GRα-A, GRα-B, GRα-C and GRα-D were observed in nasal tissues. In consistent with Sinclair’s study, previously unknown 67 kDa isoform was also identified in our study.

Previous studies on GR protein isoforms in CRS have focused mainly on total GR, GRα and/or GRβ isoforms, and the results have been controversial and inconsistent [22, 25, 26]. Pujols and coworkers revealed that GR protein expression determined by immunohistochemistry was reduced in NPs but upregulated after short-term oral GC and intranasal budesonide treatment [22]. In contrast, Watanabe and Suzaki revealed markedly increased GRα expression in inflammatory cell infiltrates in the NPs of individuals with asthma and found that the expression of GRα was significantly reduced following GC treatment [26]. In our study, GR presented different protein expression profiles either between the CRSwNP and control groups or between different endotypes of CRSwNP. We speculate that the inconsistency of the above-mentioned results could be, to some extent, that different GR protein isoforms profiles may be implicated in different responses to GC between different endotypes of CRS [3].

With regard to different translational GR isoforms, the expression levels of the GRα-A/B and GRα-C isoforms did not significantly differ between the CRSwNP and control groups or between the E-CRSwNP and NE-CRSwNP subgroups. Surprisingly, relative to the other GR isoforms, GRα-A was weakly expressed, constituting less than 3% of the total expression (the sum of the intensities of bands 1–5), although it was expected to be the predominant isoform of GR. Consistent with our findings, Saif and coworkers also reported that GRα-A, which was detectable in 57–70% of all preterm placentae, was weakly expressed, constituting less than 2% of the total expression of all isoforms [33]. This could explain in part why significantly different expression levels of GRs were not observed between diseases and responses to GC when only GRα-A was considered.

In our study, the expression of GRα-D, which was stable and abundant in each individual, was much higher in the NP group than in the control group and was much higher in the NE-CRSwNP subgroup than in the E-CRSwNP subgroup. The differences in GR isoforms among the groups may be partially attributed to possible geographic differences in different CRSwNP endotypes [1]. However, Sinclair et al. also found that patients with schizophrenia and bipolar disorder have selective increases in GRα-D isoform expression in certain brain regions [14, 36]. Isoform-specific gene regulatory profiles have been found to produce functional differences in GC-induced apoptosis in osteosarcoma cells [12]. Cells expressing GRα-C3 exhibit the highest sensitivity to glucocorticoid-induced apoptosis, while GRα-D3-expressing cells are the most resistant [12]. In one study, when the individual isoforms were expressed at similar levels in U2OS osteosarcoma or Jurkat T lymphoblastic leukemia cells, they each regulated a unique set of genes [21]. Fewer than 10% of the genes were commonly regulated by all the subtypes, indicating that the vast majority of genes were selectively regulated by different GRα isoforms [21]. These findings suggest that GRα-D, which is considered a “resistant” isoform, deserves further functional investigation in the future.

In addition, we found that the intensities of the unknown 67 kDa and 60 kDa isoforms were both drastically higher in the noneosinophilic CRSwNP subgroup than in the E-CRSwNP subgroup. Similar unknown isoforms were also found in a series of Australian studies on the expression of multiple GR isoforms among different species detected with a total GR antibody [16, 24, 33, 34]. Clifton et al. reported that translational isoforms for GRβ, GRγ, GR-A and GR-P variants might account for the presence of these unknown bands. Given that some of these unknown isoforms are also altered in relation to fetal sex and gestational age, they have some physiological activity. Furthermore, when total GR gene translation is silenced, the protein expression of these unknown isoforms is reduced along with that of the known isoforms, supporting the hypothesis that all protein bands identified by WB analysis are GR proteins. However, confirmation is required, as these unknown bands could also be degraded forms of GR [33]. Similarly, the unknown 67 kDa isoform was also found to be predominant in the DLPFC by the detection of GRα antibody [14]. Among the other splice GR isoforms, only GR-A is truncated but retains exon 9a, which contains the epitope targeted by the anti-GRα antibody in this study [14]. According to the MWs of GR proteins, the 67 kDa isoform may be a GR-A isoform, or it may be an unknown GRα translational isoform or GR-associated protein. Given its abundance in NPs, the 67 kDa GRα-related isoform could play a critical role in inflammatory signaling in NPs. However, its identity and functional properties remain to be determined [33].

Our results should be interpreted with several limitations. This study was limited by its small sample size and retrospective method. Therefore, we could not obtain separate cytosolic and nuclear protein fractions. As GC signaling is mediated mainly by GRs, a major barrier to understanding the changes in the expression of multiple GR isoforms that occur in relation to GCs is the development of glucocorticoid sensitivity or resistance in certain NP groups; thus, hormone stimulation research should be performed on NPs with different sensitivity statuses. In addition, the unknown GR isoforms and its functional properties of GR isoforms and the relationships among them remain to be further investigated.

## Conclusion

This study demonstrates that nasal tissues express multiple GR isoforms. GR isoforms presented disease and tissue-specific expression profiles either between the CRSwNP and control groups or between the E-CRSwNP and NE-CRSwNP subgroups. These data suggest the existence of a process involving coexpression of several GR isoforms that is far more complex than previously considered and indicate that GR isoforms present disease and tissue -specific expression profiles.

## Supplementary Information

**Additional file 1**. Correlationanalysis of expression level of common bands probed by anti-total GR antibody and anti-GRα specific antibody.

## Data Availability

The datasets used and/or analyzed during the current study are available from the corresponding author on reasonable request.

## Abbreviations

GC: Glucocorticosteroid
GR: Glucocorticoid receptor
CRSwNP: Chronic rhinosinusitis with nasal polyps
kDa: Kilodaltons
NP: Nasal polyp
NE-CRSwNP: Noneosinophilic chronic rhinosinusitis with nasal polyps
E-CRSwNP: Eosinophilic chronic rhinosinusitis with nasal polyps
WB: Western blot
H&E: Hematoxylin and eosin
AR: Allergic rhinitis
HPFs: High-power fields
SEM: Standard error of the mean
IQR: Interquartile range
DLPFC: Dorsolateral prefrontal cortex
