# Supplementary material for: Identification of multiple isoforms of glucocorticoid receptor in nasal polyps of patients with chronic rhinosinusitis
Source: J Otolaryngol Head Neck Surg. 2022 Jun 11;51:25. doi: 10.1186/s40463-022-00561-1 (PMC9188707; doi:10.1186/s40463-022-00561-1)
Supplement: Supplementary file 1 — Additional file 1. Correlationanalysis of expression level of common bands probed by anti-total GR antibody and anti-GRα specific antibody. [file 40463_2022_561_MOESM1_ESM.docx]

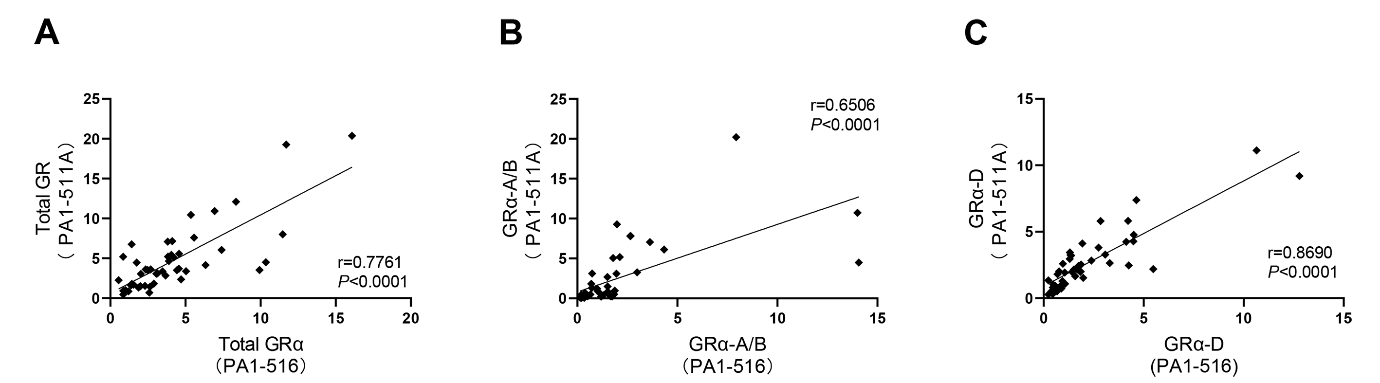


Additional file 1. Correlation analysis of the (A) the sum of expression levels for bands 1~5 and representative bands for GRs including (B) GRα-A/B and (C) GRα-D, which were detected with an anti-total GR antibody (PA1-516) and an anti-GRα antibody (PA1-516). β-Actin was used as an internal control. Abbreviations: GR, glucocorticoid receptor.
